# Heroin-Induced Leukoencephalopathy

**DOI:** 10.7759/cureus.13093

**Published:** 2021-02-03

**Authors:** Mohsen S Alshamam, Vikram Sumbly, Nso Nso, Merjona Saliaj, Dawa O Gurung

**Affiliations:** 1 Internal Medicine, Icahn School of Medicine at Mount Sinai, Queens Hospital Center, New York, USA

**Keywords:** heroin, leukoencephalopathy, chasing the dragon, ataxia, aphasia

## Abstract

Heroin-induced leukoencephalopathy (HLE) is a rare but potentially debilitating and sometimes fatal neurological disorder. Despite the widely practiced heroin use via different routes and modalities, the syndrome is said to be rare and mostly associated with inhaling rather than injecting or snorting practices. We reviewed the literature to address the latest diagnostic, therapeutic, and prognostic measures related to the condition. Here, we present a case of a 35-year-old male who admitted to inhaling heroin 18 days ago and has been experiencing ongoing neurological symptoms for the past 17 days. Imaging was consistent with extensive white matter disease at multiple levels and different anatomical regions. Although there is no known cure for HLE, the patient benefited, somewhat, from antioxidants and physical rehabilitation.

## Introduction

Leukoencephalopathy is a process that mainly affects the white matter of the central nervous system. Etiologies include infectious, toxic, inflammatory, immune-related, drug-induced, metabolic, and ischemic derangements. Leukoencephalopathy secondary to heroin inhalation is a rare condition with a poorly understood pathogenesis. The analysis of various brain images has revealed that, unlike the other causes of leukoencephalopathy, heroin-induced leukoencephalopathy (HLE) has a predilection to affect the white matter of the brain symmetrically. Histopathological specimens from autopsies of affected individuals revealed spongiform degeneration and vacuole formation of the white matter and oligodendroglia respectively. Demyelination is not common in such cases, and axons are typically spared from such damage. Symptoms can range from mild to severe, where severe cases are said to have the worst prognosis. The diagnosis is largely clinical, however, radiological diagnostics are helpful tools in forming the diagnosis. Brain biopsy is the gold standard and sufficient enough to make the diagnosis but is limited outside of autopsies. Studies have shown that patients might benefit, albeit minimally, from antioxidant therapy and physical rehabilitation.

## Case presentation

A 35-year-old male with a past medical history of hypertension and heroin abuse disorder was brought into the emergency department by his family for generalized weakness and aphasia. According to the patient's father, the patient started experiencing these symptoms 17 days ago and was forced to communicate via gestures. Additionally, due to gait instability, the patient required constant assistance to ambulate around the house. The patient's father also stated that the patient was experiencing muscular weakness more notable on the left side of the body. The symptoms started one day after the patient inhaled the fumes of heated heroin over an aluminum foil. He initially went to another facility where he was diagnosed with heroin withdrawal. He denied fever, chills, nausea, vomiting, diarrhea, sick contacts, bowel or bladder incontinence, recent travel, drug use after his symptoms started, or use of alcohol. Vital signs were within normal limits. Physical examination was noted to be positive for right beating nystagmus, apraxia with limp movements, both upper and lower extremities; dysmetria on finger-to-nose test bilaterally. The patient was also exhibiting gait ataxia, decreased grip strength in the right hand, and four of five strength in upper and lower extremities. Heel to shin could not be tested due to poor comprehension. His sensory exam was intact, and his reflexes were 2+ and symmetric throughout bilaterally. Laboratory workup was negative for diabetes mellitus (DM), human immunodeficiency virus (HIV), tuberculosis (TB), toxoplasmosis, JC virus, syphilis, coagulopathies [normal international normalized ratio/prothrombin time (INR/PT), partial thromboplastin time (aPTT) and platelet count]. Thyroid-stimulating hormone (TSH), erythrocyte sedimentation rate (ESR), and c-reactive protein (CRP) were also normal. Positive labs include lactate 2.6 (RR 0.5-2.2 mmol/L), alanine transaminase (ALT) 78 (RR 0-41 U/L). Vitamin B12 was 286 (RR 211-946 pg/mL) and ethanol level was normal. The drug panel on admission was negative (Table 1), while it was positive for opiates prior to his current admission upon symptoms’ onset (Table 2).

**Table 1:** Urine toxicology at his current admission

Drug Name	Reference Range	Result
Barbiturates	Negative	Negative
Benzodiazepines	Negative	Negative
Cocaine	Negative	Negative
Methadone	Negative	Negative
Opiates	Negative	Negative

**Table 2:** Urine toxicology at his admission to another facility prior to his admission to our hospital

Drug Name	Reference Range	Result
Amphetamine	Negative	Negative
Barbiturates	Negative	Negative
Benzodiazepine	Negative	Negative
Cannabinoid	Negative	Negative
Cocaine	Negative	Negative
Methadone	Negative	Negative
Opiates	Negative	Positive
PCP	Negative	Negative

**Figure 1:**
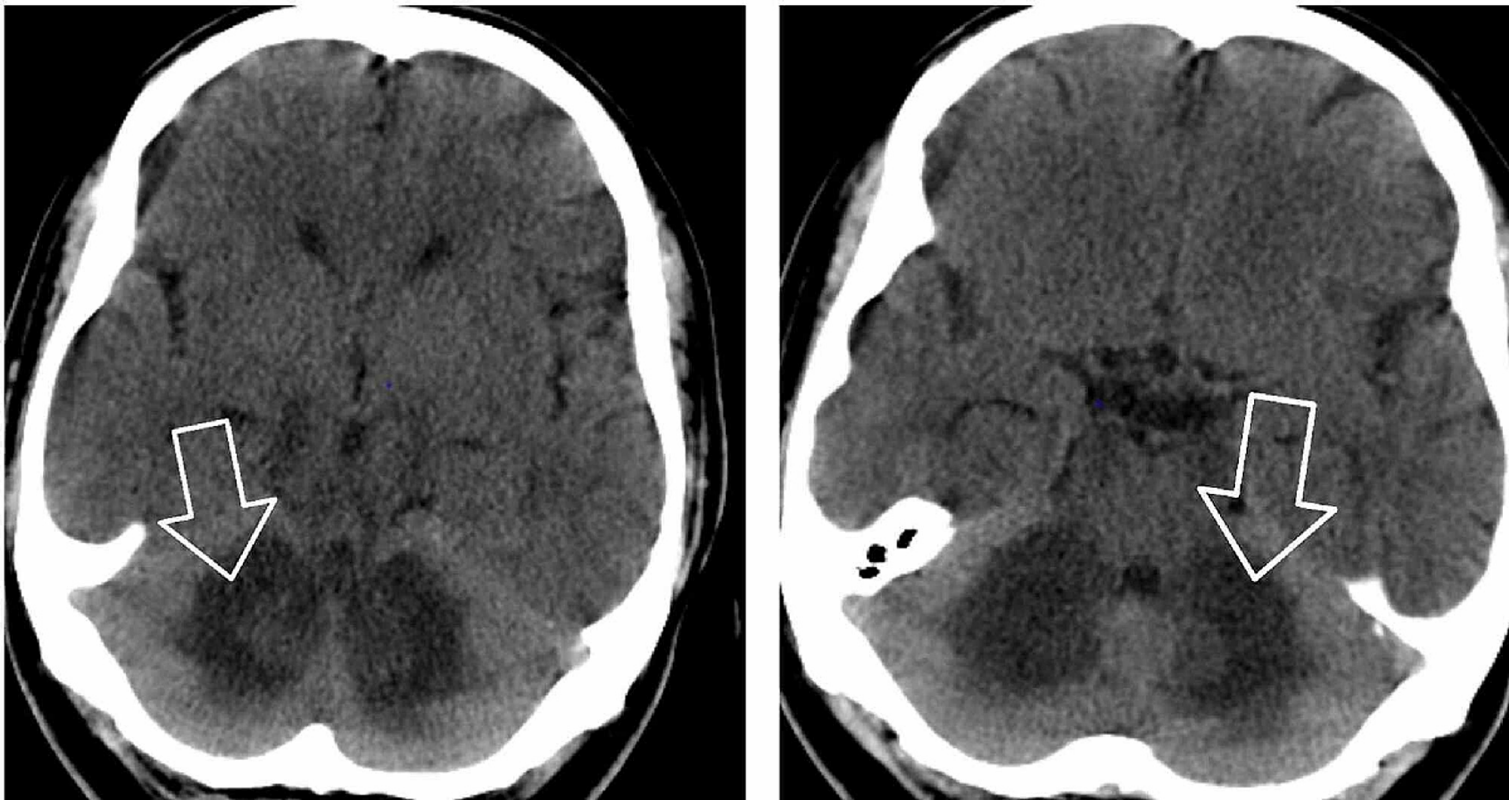
CT head without contrast

**Figure 2:**
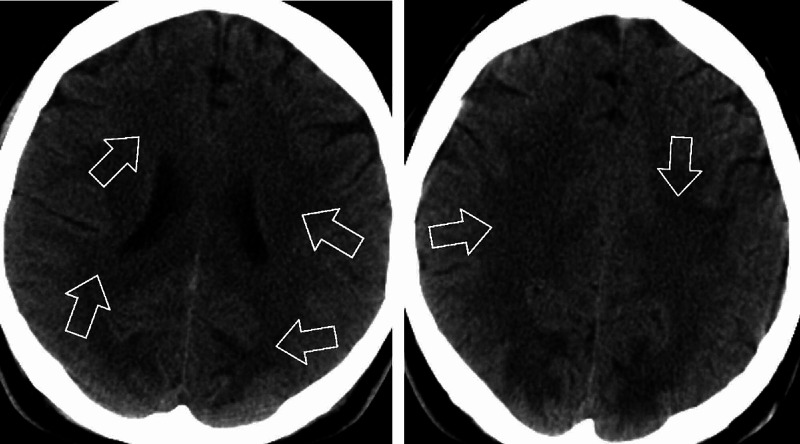
CT head without contrast

**Figure 3:**
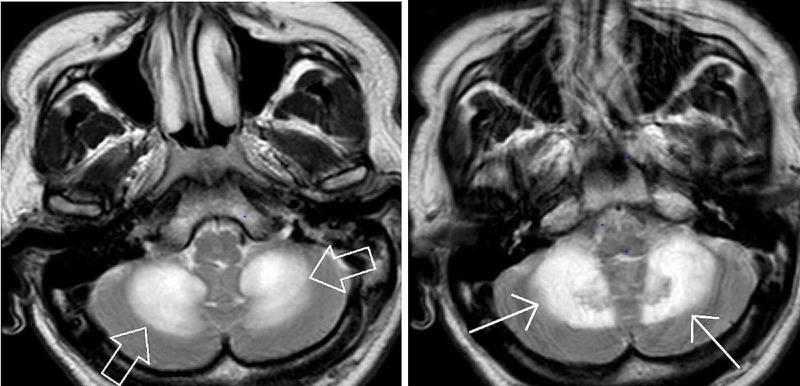
MRI brain without contrast

**Figure 4:**
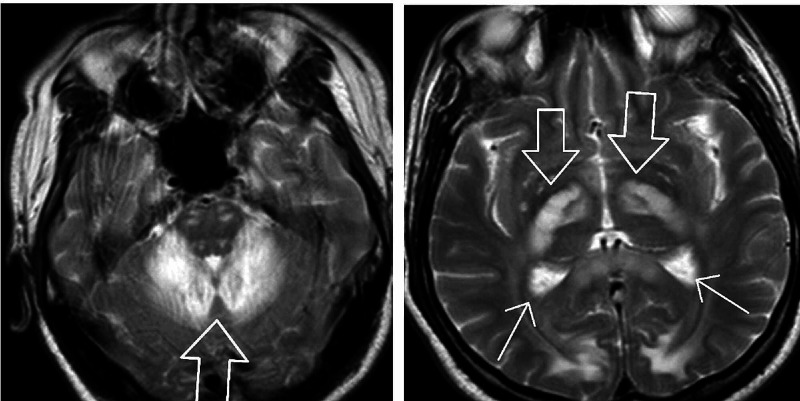
MRI brain without contrast

**Figure 5:**
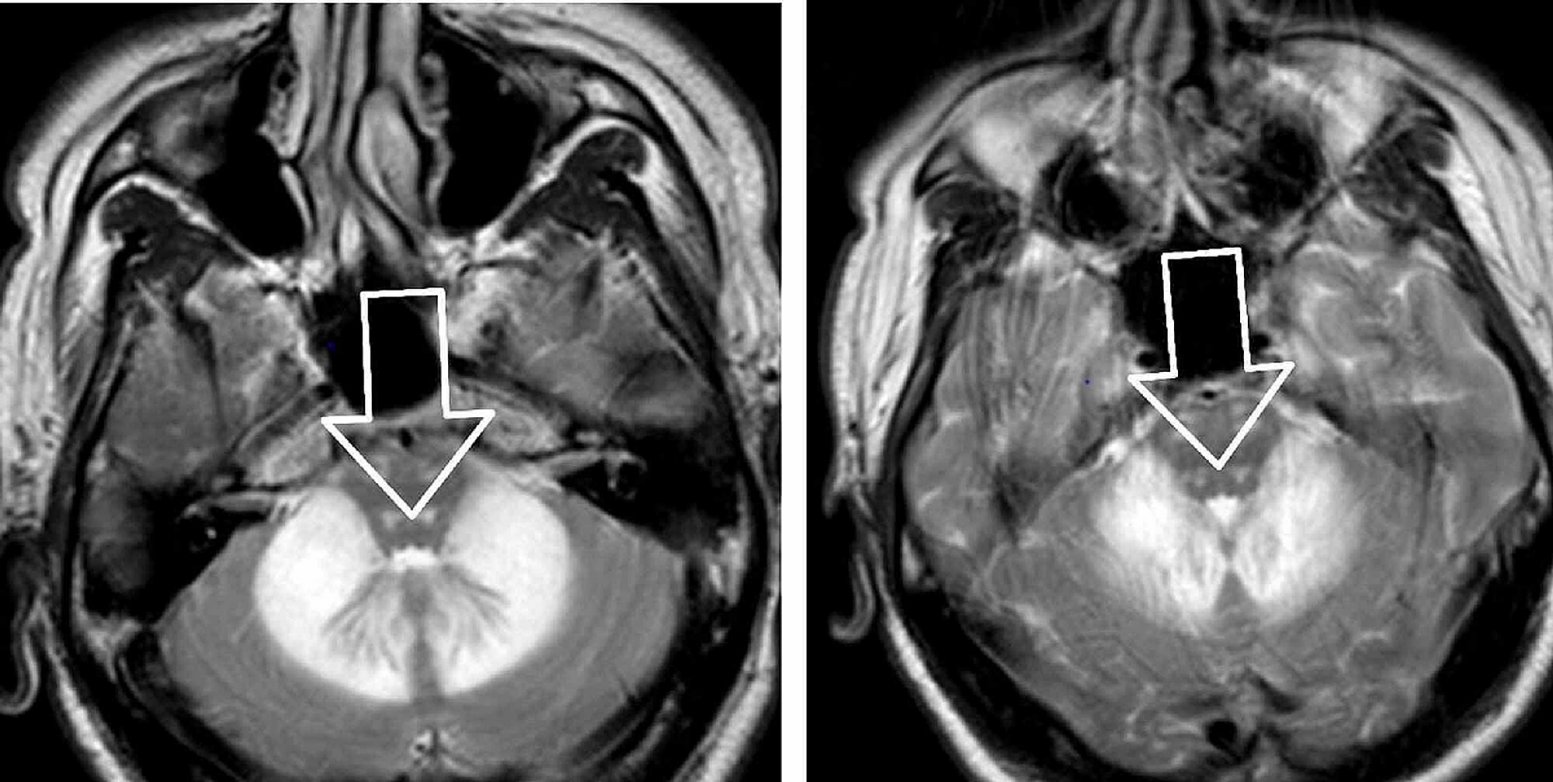
MRI brain without contrast

**Figure 6:**
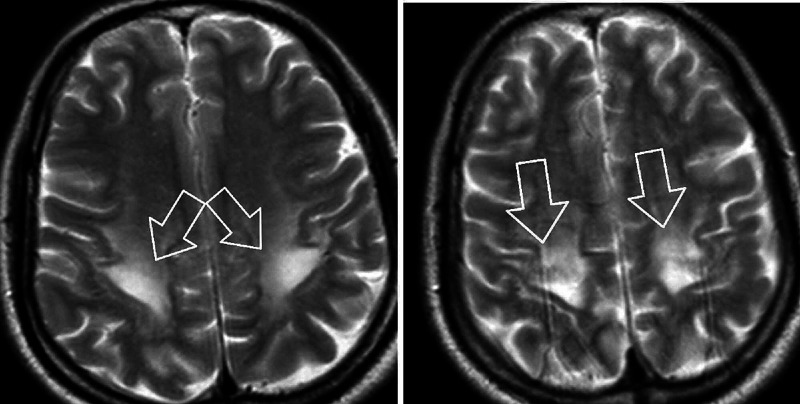
MRI brain without contrast

CT head without contrast revealed areas of abnormal low-attenuation especially involving the cerebellum, suggesting a demyelinating process with additional areas of low-attenuation also seen in the midbrain/cerebral peduncles as well as thalami and along with the posterior limbs of the internal capsule. Additional areas also include bifrontal, parietal, and especially occipital subcortical/deep white matter (Figures 1, 2). MRI brain without contrast was further pursued, which showed large bilateral symmetric areas of abnormal increased T2/fluid-attenuated inversion recovery (FLAIR) signal intensities are again seen in the left and right sides of the cerebellum/brachium pontis including dentate nuclei as well as left and right pons, midbrain, cerebral peduncles and also includes the anteromedial aspects of the thalamus, posterior limbs of the internal capsules as well as posterior corona radiata/centrum semiovale and bilateral parietal subcortical/deep white matter. Additional abnormal signal intensity also includes the splenium of the corpus callosum as well as occipital subcortical/deep white matter in a symmetric pattern (Figures 3, 4, 5, 6). Neurology was consulted who aided in the diagnosis and recommended the use of antioxidants, vitamin E, vitamin C, and coenzyme Q10; a trial of rituximab and rehabilitation. Vitamin B12 supplementation was also started. He was started on amlodipine 5 mg daily for hypertension. Throughout his hospitalization, he had little improvement in his neurological symptoms. He was discharged 10 days later so that he could receive further care at an acute rehabilitation facility.

The patient spent approximately 20 days at the acute rehabilitation facility, where he had some improvement in speech, coordination, and functional status; however, he remained severely ataxic [refer to the manual muscle test (MMT) scores tables at admission and discharge from the acute rehabilitation center (Table 3 and Table 4)]. He was discharged home with a standard manual wheelchair and home physical therapy. A 30-day follow-up revealed that the patient was able to use a walker and wheelchair despite constant left-sided hemiparesis. He maintained good bowel and bladder control and was able to enunciate words such as “ I’m good”, “water” and “I’m hungry”.

**Table 3:** MMT at admission to the acute rehab facility Manual muscle test (MMT); right upper extremity (RUE); left upper extremity (LUE); right lower extremity (RLE); left lower extremity (LLE)

Muscle Name/Action	Deltoid	Biceps	Triceps	Thumb Extension	Finger Abduction	Deep Finger Flexors/Grip
RUE	4-	4-	4-	4-	4-	4-
LUE	4	4	4	4	4	4
Muscle Name/Action	Hip Flexion	Knee Extension	Ankle Dorsiflexion	Extensor Hallucis Longus	-	-
RLE	4-	4	4	4	-	-
LLE	4	4	4	4	-	-

**Table 4:** MMT at discharge from the acute rehab facility Manual muscle test (MMT); right upper extremity (RUE); left upper extremity (LUE); right lower extremity (RLE); left lower extremity (LLE)

Muscle Name/Action	Deltoid	Biceps	Triceps	Thumb Extension	Finger Abduction	Deep Finger Flexors/Grip
RUE	5	5	5	5	5	5
LUE	5	5	5	5	5	5
Muscle Name/Action	Hip Flexion	Knee Extension	Ankle Dorsiflexion	Extensor Hallucis Longus	Plantar Flexion	-
RLE	5	5	5	4+	5	-
LLE	5	5	5	4+	5	-

## Discussion

HLE is a rare debilitating disease that affects the white matter of the brain symmetrically. As the name implies, this disease is secondary to heroin use. Although it is most commonly seen in heroin users who inhale the fumes of the heated heroin powder on a piece of aluminum foil, it can also be seen in people who inject or snort heroin as well [1-3]. Even though heroin history can be dated back to the 1950s, the disease was first reported by a cohort study from the Netherlands [4]. An unknown toxin, aluminum toxicity, and mitochondrial dysfunction are some of the proposed pathogenetic pathways, however, the exact etiology has remained undiscovered [5,6].

The syndrome is classified in terms of severity of symptoms which range from mild to severe. Mild cases mostly affect the pseudobulbar and the cerebellar functions presenting with dysarthria and ataxia, however, symptoms of hyperreflexia, weakness, and spasticity are said to represent the moderate form of the syndrome. In severe cases, stretching spasms and hypotonic paresis are common symptoms, among others, after which death can ensue. Parkinsonian symptoms or features can be seen as well, however, they are not usually confined to a specific degree of severity, and they are said to be reversible in some cases [4].

Although HLE diagnosis is largely clinical, imaging can help in the diagnostic process. The diagnostic criteria for HLE are both inclusive and exclusive. The five inclusion criteria are signs and symptoms suggestive of HLE, positive heroin toxicology, confirmation of heroin use by patient or witness, neuroimaging suggestive of HLE, and brain biopsy consistent with spongiform leukoencephalopathy, respectively [2]. The exclusion criteria comprise any of the following: a confirmed toxin or substance other than heroin, known to produce clinical findings similar to HLE; findings overtly suggestive of infectious, demyelinating, vascular, or paraneoplastic etiology; or, a predominant cortical involvement in neuroimaging with relative sparing of the subcortical and posterior fossa areas [2]. In the absence of any of the exclusion criteria, the diagnosis is definite when all of the five criteria are present, probable if the first four are met, and possible when only the first three are present [2].

On imagining, classic MRI findings include diffuse white matter hyperintensities on T2 and FLAIR sequences in the cerebellum, posterior cerebrum, and posterior limbs of the internal capsule [2]. Neuropathology from brain specimens of HLE autopsies showed spongiform degeneration (spongiform encephalopathy) of the white matter, vacuole formation in oligodendroglia, and myelin sheaths under electron microscopy, deep white matter edema, and abnormal mitochondria with the absence of inflammation. Axons are spared, whereas demyelination is said to be a non-prominent feature [5].

Although there is no definitive treatment for HLE, coenzyme Q10, vitamin E and vitamin C supplements have shown to benefit some patients [5,7-11]. The prognosis of the disease is largely dependent on the severity of symptoms. The mortality rate approaches 100% in patients within the severe group, where it would approximately be 10% in the moderately severe group. The mild severity group should not have any deaths [2].

Given that neuropathological specimens from alive patients are discouraged, our patient only meets the first four of the five inclusion criteria, while none of the exclusion criteria are present. Our patient’s neuroimaging was suggestive of HLE because it showed bilateral symmetric hyperintensities at multiple anatomical sites. His symptoms were of mild-moderate intensity. Though our patient had a modest improvement in language and physical activity over a period of four months, he still remained far below the baseline.

## Conclusions

Even though HLE is mostly seen in patients who inhale the vapor of heated heroin, it can also be seen in patients who use heroin via other routes. Regardless of the degree of involvement, the impaired functionality is almost always debilitating with very slow recovery in mild cases. HLE has no targeted pharmacological intervention other than antioxidants and rehabilitation with modest improvements. Given the wide involvement of neurological degeneration, the absence of promising treatment options, the relatively grave prognosis, and the increasing abuse of heroin, it is important that interventions should be focused on increasing social awareness of the detrimental effects of such practices.
